# Adjunctive Use of Lasers in Peri-Implant Mucositis and Peri-Implantitis Treatment: A Systematic Review

**DOI:** 10.3390/dj8030068

**Published:** 2020-07-03

**Authors:** Marianna Chala, Eugenia Anagnostaki, Valina Mylona, Anastasios Chalas, Steven Parker, Edward Lynch

**Affiliations:** 1 Department of Surgical Sciences and Integrated Diagnostics, University of Genoa, 16132 Genoa, Italy; 2 Leicester School of Pharmacy, De Montfort University, Leicester LE1 9BH, UK; eugenia.anagnostakis@my365.dmu.ac.uk (E.A.); vasiliki.mylona@my365.dmu.ac.uk (V.M.); steven.parker@my365.dmu.ac.uk (S.P.); edward.lynch@hotmail.com (E.L.); 3 Private Practice, Athens 11525, Greece; tasoschalas@yahoo.com; 4 School of Dental Medicine, University of Nevada, Las Vegas, NV 89154, USA

**Keywords:** laser, peri-implantitis, peri-implant mucositis, systematic review

## Abstract

Background: The aim of this systematic review is to compare the effectiveness of lasers in the treatment of implant mucositis and peri-implantitis compared to conventional treatment (non-surgical or surgical: resective or regenerative). Methods: Sources of PubMed, Cochrane and Google Scholar search engines were used on articles published from 1997 to 2020 in English, with selected keyword criteria applied. Nine randomized controlled trials (RCTs) were selected. Results: All included studies were considered of “high quality” according to the quality assessment scale. The comparative assessment of the RCTs was done twice for each RCT based on the type of treatment and according to wavelength. There is strong scientific evidence that, regarding non-surgical treatment, adjunct laser application can provide better results only in the short term (three months). Regarding the surgical approach, the method of decontamination plays a subordinate role. All wavelengths/applications presented similar results. Conclusion: Within the limitations of this study, the adjunctive use of lasers in the treatment of peri-implant inflammation is effective for up to three months; there is no strong evidence regarding the long term benefit compared to conventional treatment.

## 1. Introduction

The aim of this systematic review is to compare the effectiveness of the adjunctive use of lasers for the treatment of peri-implant mucositis or peri-implantitis compared to the conventional treatment (non-surgical or surgical: resective or regenerative). The widespread use of dental implants during recent decades has established them as the treatment of choice for restoring partial or full edentulism in everyday clinical practice [1,2]. Over time and despite their high survival rate and predictable outcomes, osseo-integrated implants can lose supportive bone, followed by soft tissue recession. A marginal bone loss of not more than an average of 0.2 mm after the first year of function, due to biological processes of bone remodeling, is acceptable and does not indicate any early signs of pathological conditions or inflammation [3,4]. Information through observation and investigation has shown that microorganisms and inflammation may infect the surrounding hard and soft peri-implant tissues in a similar way so that they infect the periodontium of natural teeth. Due to the reduced vascularization and parallel orientation of the collagen fibers, peri-implant tissues are more susceptible to peri-implant inflammation and this represents one of the most frequent complications that may lead to implant loss [5,6].

Peri-implant inflammation consists of two types of pathological condition, termed mucositis and peri-implantitis, but transitions between them are often fluent and not clearly clinically separable. The term “peri-implant mucositis” (PIM) is used for the microbial-induced, reversible inflammatory process in the peri-implant soft tissue only, with the clinical symptoms of reddening, swelling and bleeding on periodontal probing (BOP) [7,8,9,10]. Peri-implantitis (PI) is an irreversible disease of both hard and soft tissues surrounding the implant, together with progressive bone resorption (beyond biological remodeling of bone loss) and decreased osseointegration, increased pocket depth formation and purulence around functioning implants [7,8,9,10,11,12,13,14]. PIM has been reported to affect 80% of patients with dental implants and 50% of implants; PI appears to affect 28–56% of patients and 12–43% of implants [15].

According to many studies, it seems that a nonsurgical approach is sufficient for the management of PIM [2,16]. Mainly, this includes mechanical debridement to remove biofilm and calculus from the implant surface, with or without the adjunctive use of antimicrobials and good oral hygiene maintenance [16]. PI management remains unpredictable with no generally acceptable consensus and may require open flap surgery (resective and/or regenerative) as monotherapy, or as a second stage procedure, if the non-surgical treatment fails [2,5]. The present data indicate that non-surgical treatment is not effective alone, since only limited improvements in the main clinical parameters have been reported and there is a high possibility of disease reoccurrence [16]. More research is required to prove which therapies will result in the most predictable outcomes. The key factor in all treatment alternatives seems to be the proper method for effective decontamination of the implant surface, because in terms of surface decontamination the literature does not clearly indicate superiority of a specific decontamination protocol [2,13,16].

Lasers have been recommended for several aspects of implant dentistry, as improvement of osseointegration through photo-bio-modulation (PBM), post-operative treatment (PBM: accelerated wound healing and analgesic effect), second stage surgery for implant recovery, implant bed preparation, sinus lift procedures, and PI treatment (implant surface decontamination and implant surface modification) [17,18,19,20,21,22,23,24,25,26,27,28,29,30,31,32,33,34,35,36,37,38,39,40]. The laser effect on tissue (laser-tissue interaction) is wavelength and energy dependent. All laser wavelengths with relatively high fluence can be used to remove (ablate) infected granulomatous tissues around implants and at the same time reduce the microbial load in the treated sites together with good hemostasis, but only erbium family lasers are recommended for treating exposed bone during bone-defect debridement in open flap surgery [41]. Lower levels of energy can stimulate tissues and cells without producing irreversible changes (PBM), thus promoting wound healing [42]. During laser surgery, PBM effects may be induced in tissue adjacent to the surgical site due to photon scatter gradient effects over distance [43,44].

Lasers can be used for decontamination of the implant surface. Titanium absorbs irradiation produced from infrared and mid-infrared laser wavelengths such as diodes, Nd: YAG and the erbium family. Carbon dioxide (CO_2_) (far-infrared) irradiation is mainly reflected (>90%), but there is always the risk of a temperature rise in case of high energy delivery [45,46]. Absorption results in heat production, which is an undesirable effect, since it may cause surface alterations (melting and cracks) and damage the surrounding tissues. Due to their high peak power, Nd: YAG lasers are not recommended for decontamination of implant surfaces (risk of partial melting, cracking, and crater formation), irrespective of the power output [25]. The diode laser does not damage the titanium surface and it is capable of decontaminating rough implant surfaces, though it also has the risk of heat generation on peri-implant bone tissue when used with improper irradiation parameters and techniques [47,48]. Er: YAG and Er, Cr: YSGG (2780 nm) lasers, when used with constant water irrigation and appropriate irradiation, cause no visible changes on titanium surfaces with minimum temperature elevation [49,50,51,52]. Another way to disinfect the implant surface is via the use of antimicrobial photodynamic therapy (aPDT). This involves administration of a non-toxic dye (photosensitizer) inside the peri-implant pockets, followed by illumination using light of an appropriate wavelength which, in the presence of oxygen, leads to the formation of reactive oxygen species that causes microbial cell death [53,54,55,56,57,58,59,60].

## 2. Materials and Methods

### 2.1. Search Strategy

An electronic research was carried out through the PubMed, Cochrane Database and Google Scholar data bases between 25 March and 10 April, and 10 June and 17 June, 2020.

The following terms were used as keywords:

(Laser or diode or Nd: YAG or Er: YAG or Er, Cr: YSGG or CO_2_ or photodynamic therapy or photo-bio-modulation) and (peri-implant mucositis or peri-implantitis or periimplantitis or peri implantitis). An initial search without grouping of key words yielded a total of 2573 items, but with keyword grouping this was reduced to 326 and this number was further filtered.

The search identified 326 articles.

With successive filtering applied (no anecdote, incomplete, case studies, etc.) only human RCTs in the English language, within the last 15 years, were included. The remaining articles totaled 37.

The following criteria were applied for further inclusion into the systematic review.

- RCTs;
- At least single blinding applied;
- At least 10 patients with one or more implants each;
- Type of inflammation: mucositis or peri-implantitis and criteria of diagnosis required;
- Laser used in test group;
- Interventions: the test groups received laser therapy additional to conventional treatment and the control groups received conventional treatment only;
- Follow up: at least three months.

Exclusion criteria:

- Studies using LEDs as the light source (only applicable to aPDT studies);
- Studies without a control group.

**Figure 1:**
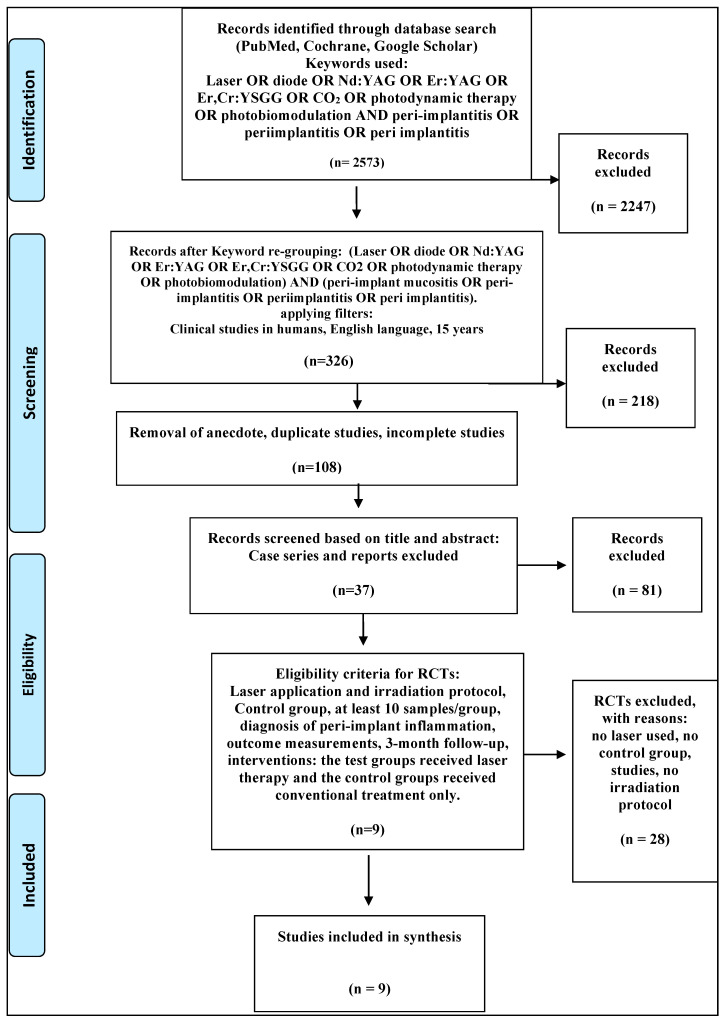
PRISMA flow-chart - selected criteria for the included studies.

According to the above, nine studies were selected in this systematic review. The flowchart of this process was in accordance with PRISMA guidelines and is shown in Figure 1 [61].

### 2.2. Data Extraction

From the nine selected studies, the following evidence was extracted:

- Publication details (authors, year of publication);
- Type of peri-implant inflammation/diagnosis;
- Treatment approach (non-surgical/surgical: regenerative or resective);
- Number of patients/groups of study;
- Number and type of implants;
- Method of implant surface decontamination;
- Wavelength and irradiation protocol;
- Follow-up;
- Bleeding on probing;
- Plaque index;
- Probing depth;
- Clinical attachment level;
- Gingival recession;
- Bone level.

### 2.3. Quality Paper Assessment

The eligibility criteria according to the PICOS [62] process have been interpreted as follows:

- Population = adults with peri-implantitis or peri-implant mucositis;
- Intervention = Mechanical debridement + Laser (both in surgical and non-surgical modalities);
- Compared with = Mechanical debridement alone (both in surgical and non-surgical modalities);
- Outcome of interest = Pain; Healing; probing pocket depth (PPD), bleeding index (BI), etc.;
- Study type = Randomized Controlled Trials.

Furthermore, for the selection of eligible articles, a grade scale for quality assessment was applied based on the following criteria:

- Randomization and blinding;
- Comparability of groups at baseline (e.g., severity of disease);
- Description of treatment and irradiation protocol;
- Clinical measurements at baseline and at follow up;
- Radiographic evaluation at baseline and at follow up.

The classification was performed according to the number of positive answers to the above questions.

(1) High quality: 4–5
(2) Medium quality: 2–3
(3) Low quality: 1

### 2.4. Scientific Evidence

The scientific evidence has been assessed on the basis of studies with equal quality. The level of evidence has been set according to the following presented criteria:

- Strong scientific evidence: the conclusion is corroborated by at least two studies;
- Contradictory scientific evidence: the conclusion is corroborated by studies whose findings contradict each other.

## 3. Results

### 3.1. Primary Outcome

The primary goal of this systematic review was to evaluate the treatment outcomes of the included studies and critically appraise their results.

### 3.2. Quality Assessment

Based on the above mentioned grade scale, all the included studies were assessed as “high quality” studies with scores of 4 or 5 [63,64,65,66,67,68,69,70,71]. One study was graded 4 [70], due to missing radiographic data during the follow-up.

### 3.3. Data Presentation

The analyzed respective data are shown in Table 1, Table 2 and Table 3.

#### 3.3.1. Comparative Assessment per Each Pathological Condition (Figure 2)

a. Mucositis.

Two RCTs: Aimetti et al. [63], Sánchez-Martos et al. [64].

Aimetti et al. [63] concluded that the adjunct use of a laser did not yield any statistically significant clinical benefit as compared to conventional treatment at three months (differences between both groups *p* = 0.651 and *p* = 0.548, for site level and implant level respectively).

Sánchez-Martos et al. [64] concluded that the adjunctive use of a laser for PIM treatment was more effective in reducing bleeding on probing (BOP) at three months (*p* < 0.05 between groups).

According to the above, there is contradictory evidence regarding the beneficial effect of a laser as an adjunct compared to conventional mucositis treatment at three months follow-up [63,64].

b. Non-surgical PI.

Three RCTs: Renvert et al. [65], Abduljabbar et al. [66], Romeo et al. [67].

Renvert et al. [65], did not provide measurements at three months. Abduljabar et al. [66] reported the plaque index (PI) (*p* < 0.05), bleeding on probing (BOP) (*p* < 0.05) and pocket depths (PD) (*p* < 0.05) between groups at three months. Similarly, Romeo et al. [67] reported better clinical parameters at three months, but no *p*-values were available.

Regarding the final treatment outcome at six months, Renvert et al. [65] reported *p* = 0.22 for BOP, and *p* = 0.55 for PD compared to the control group. Abduljabbar et al. [66] reported *p* > 0.05 for PI, BOP, and PD between the groups.

Therefore, there is strong scientific evidence that, regarding non-surgical treatment, adjunct laser application can provide some better results after three months, but no significant improvement after six months compared to conventional treatment [65,66,67].

c. Surgical PI.

Four RCTs: Schwarz et al. [68], Deppe et al. [69], Papadopoulos et al. [70], and Albaker et al. [71].

Open flap resective therapy:

According to Papadopoulos et al. [70], both groups had similar clinical outcomes and the laser offered no additional benefit after six months. This is in agreement with Albaker et al. [71] at both the six- and twelve-months follow-up periods.

Deppe et al. [69] evaluated clinical attachment levels (CAL) and radiographic distance from the implant shoulder to the first bone contact (DIB) values at four months and five years. They reported that CAL in residual bone was significantly better (*p* < 0.05) at both time intervals.

Regarding DIB, a significant difference was shown at five years for residual bone.

According to Papadopoulos and Albaker [70,71], there is strong scientific evidence to support that the laser effect is not beneficial after six months. This is in partial agreement with Deppe et al. [69] who could not report statistically significant changes between the groups after four months (except for CAL), but did show significantly better results for the test group after five years.

Open flap regenerative therapy:

Deppe et al. [69] reported that for augmented bone, a statistically significant difference was observed only after four months.

Regarding DIB, a significant difference was shown at four months for the augmented bone.

They concluded that the decontamination method played a subordinate role after five years of follow-up. This is in total agreement with Schwarz et al. [68], who reported that after seven years the outcome was not related to the initial method of implant surface decontamination.

To summarize, there is strong scientific evidence that both treatments (mechanical debridement or mechanical debridement and laser surface decontamination, followed by guided bone regeneration (GBR)) result in the same outcome in the long-term [68,69].

#### 3.3.2. Comparative Assessment per Laser Wavelength/Type of Application (Figure 3)

Studies with Nd: YAG [66] and CO_2_ [69] are not included as they are single studies for the particular wavelength.

a. Diode lasers in peri-implant inflamed tissues.

Three RCTs: Aimetti et al. [63], Sánchez-Martos et al. [64], and Papadopoulos et al. [70].

Aimetti et al. [63] did additional PBM after treatment. This study had the highest number of implants. They reported no statistically significant difference between the groups after three months (all *p*-values > 0.05). This was in agreement with Papadopoulos et al. [70].

On the other hand, this finding was not confirmed by Sánchez-Martos et al. [64], who concluded that adjunct laser treatment resulted in statistically significant improved clinical values after three months follow-up compared to the control group (*p* < 0.05).

Therefore, there is contradictory evidence regarding the effect of diode lasers in peri-implant inflamed tissues at three months [63,64,70].

b. Er: YAG lasers in peri-implant inflamed tissues.

Two RCTs: Renvert et al. [65], and Schwarz et al. [68].

Renvert et al. [65], reported similar clinical findings in both groups after six months (no statistically significant difference between groups, *p* = 0.84) and limited overall clinical improvement. Respectively, Schwarz et al. [68] reported a similar clinical outcome after seven years in both groups (no *p*-values available) and that the outcome was not relevant to the implant surface decontamination procedure.

According to the above, there is strong scientific evidence that the Er: YAG laser does not offer a significant benefit in the clinical outcome after six months or seven years [65,68].

c. aPDT application in peri-implant inflamed tissues.

Two RCTs: Albaker et al. [71], and Romeo et al. [67].

Romeo et al. [67] reported better clinical outcomes at three months follow-up for the aPDT group compared to the control (no statistical analysis, no *p*-values available). Albaker et al. [71] reported no additional benefit of aPDT after the six- and twelve-months follow-up (no significant difference between groups over time *p* > 0.05 in all parameters examined).

None of the results can be confirmed since there were no clinical measurements from Albaker et al. [71] after three months and there was no statistical analysis from Romeo et al. [67].

## 4. Discussion

Peri-implant mucositis is the precursor to peri-implantitis, as is gingivitis for periodontitis. A continuum exists from healthy peri-implant mucosa to peri-implant mucositis to peri-implantitis. Prevention of peri-implant mucositis may prevent conversion [72].

Peri-implant mucositis is an inflammation in the soft tissues which is a reversible host response to periodontal pathogens. This can progress to peri-implantitis, regarded as a destructive inflammatory process of soft and hard tissues surrounding a dental implant and typically leads to bone and possible implant loss [73].

Although no evidence for a single protocol or recommendation is available, the treatment of peri-implantitis will draw upon the following aims:

1. Assessment of etiology
2. Assessment of implant survivability
3. Elimination of biofilm and debridement
4. Re-establishment of biocompatibility
5. Re-osseointegration as required
6. Re-establishment of function
7. On-going review and maintenance

The many treatment modalities investigated over three decades have failed to establish an ideal predictable therapy, nor has any monotherapy been established [74]. Laser-assisted therapy has in consequence grown in application.

In the current literature, there is a high number of published clinical studies reporting the adjunctive use of lasers in combination with conventional debridement in the treatment of peri-implant inflamed tissues with promising results [38,75,76,77,78,79,80,81,82,83,84,85,86]. The findings of the present systematic review are in agreement with the above-mentioned literature. One of the main limitations of this systematic review was that the large variety of irradiation protocols, together with the missing reported parameters, does not allow any comparison, let alone a quantitative synthesis of the data from the included studies. The same problem was highlighted by two other systematic reviews and meta-analyses on the same subject [87,88]. In one of these, it is not scientifically accurate to compare the efficacy of different laser wavelengths under the generic term laser treatment, because this may lead to misleading conclusions [75]. On the other hand, comparing the effect of one laser wavelength on different degrees of inflammation, without taking into account the type of treatment, can also be misleading. In recognition of the above, in this systematic review the comparative assessment of the studies was done in two ways: according to severity and type of treatment and according to laser wavelength/type of application. The weakness of comparing evidence and not proceeding to a meta-analysis is due to the substantially varying conventional non-surgical or surgical treatment protocols, the heterogeneity regarding disease severity and treatment suggested, the wide variety of the irradiation protocols and the high number of studies performed by the same group of authors in comparison with the overall limited number of RCTs. For the purpose of clarity, “non-surgical” and “surgical” treatments are considered in relation to whether a surgical mucogingival soft tissue flap is raised or not (latter vs. former).

### 4.1. Peri-Implant Mucositis

For PIM treatment, mechanical debridement alone involves the supra and subgingival debridement of the implant surface. The main objective is to remove peri-implant biofilm and calculus without altering the implant surface, with the goal of re-establishing a healthy peri-implant mucosa [16]. In this study, two papers were chosen and reported the use of diode lasers of 810 nm and 980 nm wavelength; any superiority of the laser adjunctive therapy was not confirmed by the findings of the included studies [63,64].

### 4.2. Non-Surgical Treatment for PI

With an increase in the complexity of the disease process, non-surgical treatment of PI may offer some challenge in terms of surgical access. Complete resolution of the inflammation, or total inhibition of the progressive nature of PI, is not reported by any of the included studies [63,64,65,66,67,68,69,70,71]. The laser wavelengths identified in the selected papers as used were the Diode 670 nm, Nd: YAG 1064 nm and Er: YAG 2940 nm. Renvert et al., using the erbium laser, reported that, if defining a positive outcome as having a PD > 5 mm with a BOP and suppuration at baseline, but no PD < 5 mm, no BOP and no suppuration at 6 months, none of the cases in either group obtained this level of treatment outcome [60]. Such findings are at variance with the findings of Al-Falaki et al., demonstrating that treatment resulted in the resolution (<4 mm) of 91% of the sites after six months [89].

Abduljabbar et al. [66], with the Nd: YAG laser, reported no statistically significant difference in crestal bone loss (CBL) amongst patients between groups at the three- and six-month follow-ups compared with baseline. In both groups, CBL was approximately 2 mm at all time intervals, which is considered normal due to bone remodeling. In addition, peri-implant plaque index, BOP and PD were significantly lower amongst patients in the test group (mechanical debridement MD + Nd: YAG laser) compared with patients in the control group (MD alone) at three-month follow-up. Therefore, peri-implant soft tissue healing was significantly faster when MD was performed with an adjunct Nd: YAG laser compared with MD alone. They related this result to the possibility that adjunctive laser decontamination was more effective in reducing the counts of pathogenic microbes as compared to when MD was performed alone, and to the possible PBM effect [66], since it has also been reported that MD with adjunct single application of Nd: YAG laser irradiation can reduce the expression of proinflammatory cytokines in the gingival crevicular fluid of patients with periodontal disease [90]. Nevertheless, this is a short-term effect (three months).

The superiority of implant surface laser decontamination is not confirmed by other studies, supporting that lasers did not show additional advantages over traditional systems, and even rinsing with saline has shown a successful outcome [69,91,92]. According to another systematic review, the benefit of using laser treatment during a non-surgical approach should be investigated as a prequel to surgical treatment [75]. Based on the studies included in the present systematic review, non-surgical therapy was efficient at controlling peri-implant inflammation for six months post-intervention [65,66,75,81].

The effect of the two-step treatment may delay the disease process, since it resulted in the longest delay of implant loss (6.5 years, on average) [84]. Perhaps a two-step approach, repeated interventions using the same or different laser wavelength or laser application, would be able to maintain or improve the long-term result. This is in accordance with the Third European Association for Osseointegration (EAO) Consensus Conference 2012 recommendations: a regular maintenance program may be needed for the long-term management of peri-implantitis lesions after non-surgical interventions [93].

### 4.3. Surgical Procedures for PI

#### 4.3.1. Resective Approach

Open flap procedures include resective and regenerative treatment. Prior to surgical therapy, local and systemic risk factors, such as poor oral hygiene, smoking, and periodontitis, should be under control [93]. From the studies included only one [69] included edentulous patients, and the others performed conventional mechanical debridement on the whole dentition prior to surgery to reduce the total bacterial load [68,70,71]. Different reported findings from studies using the resective surgical approach are expected, because they are influenced by factors not yet fully understood but which may draw upon variations in etiology, pathology and surgical technique [93]. Figuero et al. support that this surgical intervention aims to eliminate the inflammatory changes responsible for the disease process and maintains the position of the soft-tissue margin around the implant neck. This can only be attained when the peri-implant bone loss is shallow [16]. This is not in accordance with Deppe et al., who suggested that CO_2_ laser decontamination may be more efficacious than conventional decontamination in deep, narrow bony defects and especially when combined with soft tissue resection [69]. These authors reported that four months after treatment laser-assisted decontamination combined with soft tissue resection resulted in DIB values very similar to those yielded by conventional decontamination plus soft tissue resection, together with better CAL. At the long-term follow-up (five years), ongoing bone resorption was observed only in the conventionally decontaminated group. The laser assisted decontamination group presented stable DIB values over time [69]. Papadopoulos et al. [70] reported a similar pattern of PPD reduction in both groups compared to baseline. CAL reduced significantly after surgery and increased gradually in both groups during the observation time, but only in the test (laser) group did CAL present a significant improvement at the three- and six-month time points in comparison to the baseline (*p* < 0.05) [70]. Perhaps this is a result of a collateral PBM effect after diode laser application, similar to the findings of Abduljabbar et al. [66]. Although a vertical gain in bone level is not expected from this resective procedure, DIB measurements would be valuable in order to establish if the gain in CAL is only due to creeping re-attachment alone, or together with an amount of bone infill.

Albaker et al., reported that PD were reduced in both treatment groups from 5.2 mm to 3.9 mm in the PDT group (*p* < 0.05) and 5.4 mm to 4.1 mm in the open flap debridement (OFD) group (*p* < 0.05) [71]. The marginal bone loss (MBL) significantly reduced further during the one-year observation period with no significant difference between the groups over this time. This finding is not in accordance with Abduljabbar et al., who reported stability of the CBL at all follow-up intervals [66].

#### 4.3.2. Regenerative Approach

According to Figuero et al., there was no evidence to recommend the use of a specific regenerative surgical technique, such as grafting with autogenous or xenogeneic grafts or bone substitutes [16]. Schwarz et al. reported that mean PD values at seven years were markedly reduced in both groups, but these changes were more pronounced at CPS (plastic curettes + cotton pellets + sterile saline) treated sites (median changes—CPS: 2.15 vs. ERL: 1.20 mm) compared to the ERL (Er: YAG) group [68]. Both treatments resulted in a marked reduction in the mean marginal recession (MR) values, with significant CAL gain throughout the seven-year follow-up. Creeping attachment was more pronounced in the ERL group. Deppe et al., on the other hand, reported statistically significant different DIB values in both augmented groups at four months, but not at five years [69]. This probably indicates a gradual loss of the augmented material over time. Schwarz et al., after healing periods of eight months and up to 6.5 years, reported that most of the implant sites investigated were associated with a new hard tissue fill in the former Class I defect area and that the large variations in the mean PD and MR changes over time might, at least in part, be attributed to the complex events of wound healing, maturation and re-modelling in Class I and Class II defect components after therapy [68]. This was in total accordance with Sinjab et al., supporting that the morphology of bony defects determined the healing potential of regenerative therapy [2]. Deppe et al. [69], comparing their findings with another study [94], reported a lesser reduction of the defect depth (about 30% to 40%), and suggested that autogenous bone can lead to more favourable augmentation of peri-implant defects than synthetic materials over the long term. Both studies, Deppe et al., and Swartz et al., suggested that, with respect to the results of augmentation procedures, the method used for decontamination seems to play a subordinate role [68,69].

### 4.4. Ideal Reporting of Irradiation Protocols

A further aspect of analysis of treatments involving the use of lasers relates to the overall concept of dose. The principal outcome of laser photonic energy application in the studies chosen is photo-thermolysis and its ability to decontaminate bacteria, incise and ablate tissue, as assist in coagulation of blood, plasma and crevicular fluid. Avoiding excess dose may predispose to unwanted effects and in general the key to the type of biological outcome in post-treatment living tissues lies in the irradiation parameters used. When referring to an irradiation protocol, and in order to compare its effectiveness with another, all the following data should be described in full detail [95]:

- Intrinsic properties such as laser manufacturer, mode, type of laser, wavelength, delivery system, emission mode, energy distribution and energy delivery.
- Adjustable parameters such as pulse width, average power, pulse repetition rate, on-off- time or continuous mode, tip to tissue distance or in contact mode, focus or defocused mode, beam divergence, fiber or spot diameter at focus, length of treatment and speed of movement.

The adoption of pre-set manufacturer’s recommended parameters may be difficult to correctly address the racial, skin-type and biotype of the patient and parameters such as average or peak power, average or peak power density, total energy, energy per pulse and energy density with movement can be calculated and allow comparison. The amount of water or air used as a coolant and the method of delivery should also be mentioned, as well as any tissue relaxation time during treatment, because they may result in a different effect on the target tissue or in this case the implant surface.

The emerging philosophy of concomitant PBM effects and especially with the use of visible and near infra-red wavelengths may further enhance the benefits of laser adjunctive use in the treatment of peri-implant pathology.

It is not possible to fully resolve the many variables in defining appropriate treatment or evaluating outcomes through a limited number of studies. It is hoped, that with the adoption of greater accuracy in applying laser photonic energy, the overall success rate of laser use may improve.

## 5. Conclusions

Within the limitations of this study, it may be concluded that the adjunctive use of lasers in the treatment of peri-implant inflammation does not offer any additional benefit compared to conventional treatment after six months; there is no strong evidence regarding the long-term benefit compared to conventional treatment.

## Figures and Tables

**Figure 2:**
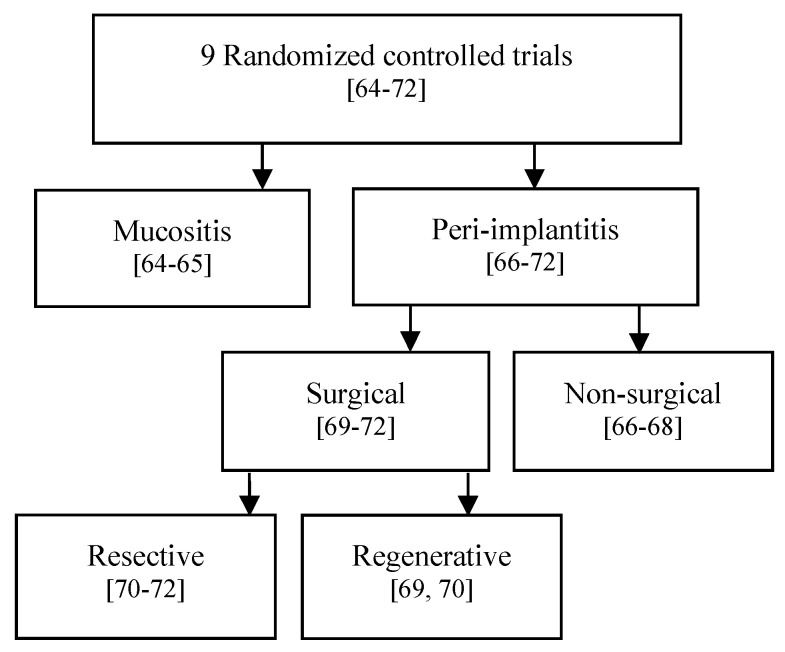
Comparison assessment of the studies according to indication.

**Figure 3:**
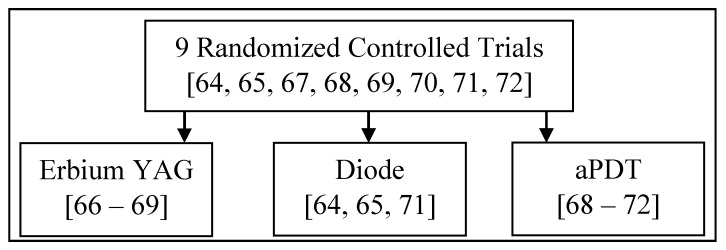
Comparison assessment of the studies according to wavelength.

**Table 1:** Analysis of peri-implant mucositis.

Authors Number of Implants/Patients	Laser/Irradiation Protocol/Treatment Protocol	Type of a/Implant b/Characteristics of Surface/Decontamination-Smoothening	BOP/PI CAL/PD	Outcome Follow Up	Comment
Aimetti et al. (2019) [63] RCT 220 implants/220 pts.	Diode (DL) 980 nm. Non-surgical approach Group 1: Debridement curettes/US Group 2: Debridement curettes/US DL + 3% H_2_O_2_ for 10 s.	a/Not mentioned b/Group 1: Mechanical debridement, Group 2: Mechanical debridement + Laser decontamination + Peroxide.	Peri-implant mucositis (PD ≥ 4 mm) BOP/PD/PI measured.	1 mth—BOP ↑ DL group (*p* < 0.01).3 mths—both groups ↓ BOP (*p* > 0.05), PI (*p* < 0.001) and PD (*p* < 0.001).	DL no statistically significant clinical benefit at 3 mths. Complete resolution obtained 38/110 (34.5%) implants in test group cf 34/110 (30.9%) implants in control group.
Sánchez-Martos et al. (2020) [64] RCT 68 implants/68 pts.	Diode (DL) 810 nm. Group 1: Debridement + CHX + 0.05% cetylpyridinium chloride (Control) Group 2: Debridement + CHX + 0.05% cetylpyridinium chloride + DL (Laser Group).	a/3i (62) int. hex/screwed abutments. Straumann (6). b/DL AP 1.0 W (Gated)/30 sec/surface with 1 cm diffuser. 300 μ tip into sulcus for 30 secs.	Mucositis. Gr 1 av. BOP of 1.176 ± 0.700, 0.264 ± 0.220 (6 wks), 0.568 ± 0.282 (3 mths). Gr 2 BOP 1.175 ± 0.795, 0.148 ± 0.150 (6 wks), 0.264 ± 167 (3 mths). Stat. sig. 95% (t-Student *p* = 0.001) between groups at 3-month. Gr 1 av PI 0.676 ± 0.374, 0.588 ± 0.526 (6 wks), 0.509 ± 0.370 (3 mths). Gr 2 av. 0.824 ± 0.541, 0.248 ± 0.3155 (6 wks), 0.480 ± 0.336 (3 mths). Stat. sig. 95% (t-Student *p* = 0.041) groups at 6 weeks. CAL No stat. sig. 95% throughout the study. Gr 1 PD 1.303 ± 0.409 mm, 1.137 ± 0.222 mm (6 wks), 1.166 ± 0.263 mm (3 mths). On the other hand, the Gr 2 1.277 ± 0.347 mm, 0.833 ± 0.374 mm (6 wks), 1.068 ± 0.103 mm (3 mths). Stat. sig. 95% (t-Student *p* = 0.041) 6 weeks.	A better response of the gingival index was obtained, especially in bleeding on probing, which avoids a significant decrease of the inflammation in the peri-implant tissues.	The use of diode laser as an adjunctive therapy to the conventional treatment of peri-implant mucositis showed promising results, being more effective reducing the inflammation of the peri-implant tissue, positioning itself as a valuable tool for the treatment of peri-implant pathologies.

**Table 2:** Analysis of peri-implantitis, non-surgical approach.

Authors Number of Implants/Patients	Laser/Irradiation Protocol/Treatment Protocol	Type of a/Implant b/Characteristics of Surface/Decontamination-Smoothening	BOP/PI CAL/PD	Outcome Follow Up	Comment
Renvert et al. (2011) [65] Blinded RCT 86 implants—42 patients Non-surgical.	Er:YAG 2940 nm. Grp 1: Perio Flow Device, Grp 2: Er:YAG 100 mJ/pulse/10 Hz. Fluence 12.7 J/cm^2^.	a/Air -abrasive Group: machined surface: 29, medium rough surface: 16. Laser Group: machined surface: 41, medium rough surface: 14. Instrument tip was used in a parallel mode using a semi-circular motion around the circumferential pocket area of the implant.	Peri-implantitis. BOP ↓ sig. in both groups (*p* < 0.001). No differences in changes of BOP by study intervention groups (*p* = 0.22). 6 mths ↓plaque at implants in air-abrasive group (*p* < 0.05). CAL—Not measured PD ↓ in laser group 0.8 mm (SD ± 0.5), PD ↓ in air-abrasive group 0.9 mm (SD ± 0.8). No differences in PD by study group intervention (*p* = 0.55).	PI results of therapy at 6 mths similar Er:YAG or air- abrasive for debridement of implants. Both methods ↓ PD and BOP. The overall clinical improvement was limited.	No sig. diff. PD > =5 mm, BOP and suppuration at 6 mths. No sig diff in alveolar bone at 6 months in both groups.
Abduljabbar et al. (2017) [66] RCT 63 pts/79 implants Non-surgical.	Nd: YAG 1064 nm 60 to 120 s. Av *p*. 4 W 80 mJ 50 Hz. Pulse width 350 msecs + air/water.	a/Platform-switched with moderately rough surfaces b/Grp 1 = MD only, Grp 2 = MD with 1 appl Nd: YAG laser.	Peri-implantitis: BOP at >30% of PI sites, PD ≥ 4 mm and/or ≥ 3 mm bone loss/implant. 3 mths BOP ↑ MD + Nd:YAG Grp cf MD Grp. 6 mth BOP comparable in both groups. 3 mth PI ↑ MD + Nd:YAG Grp cf MD Grp. 6 mth PI comparable in both groups. CAL not measured.	No statistically significant difference in CBL among patients in groups 1 and 2 at 3- and 6-month.	Nd:YAG + MD non-surgical more effective in PI cf MD but not maintained at 6 mths. Soft tissue healing sig faster MD + Nd:YAG cf MD.
Romeo et al. (2016) [67] RCT 40 pts/123 implants Non-surgical.	DL 670 nm + MBO. Fluence 25.54 J/cm^2^, Total energy 1592 J/cm^2^.	a/Not mentioned b/Grp 1: MD Group 2: MD +aPDT.	Peri-implantitis: BOP, PD ≥4 mm, and suppuration. BOP ceased Grp 2 at 24 wks. PI Grp 2 17% at 24 wks. Control PI of 25%. No sig. diff. between grps. CAL Not measured PD Grp 2 (MD and aPDT) better with av. 2 mm cf Grp 1(3 mm). The readings remained constant at 24 weeks.	The results obtained in this study suggest that photodynamic therapy could be considered an effective method for bacterial reduction on implant surfaces.	Group 2 showed after 24 weeks a better value in terms of PD, BOP, and PI, with an average pocket depth value of 2 mm, if compared with group 1 (3 mm).

**Table 3:** Analysis of peri-implantitis, surgical approach.

Authors No. of Implants/Patients	Laser/Irradiation Protocol/Treatment Protocol	Type of a/Implant b/Characteristics of Surface/Decontamination-Smoothening	BOP/SBI (Sulcus Bleed)/PI CAL/PD/DIB (Implant Shoulder to Bone)	Outcome Follow Up	Comment
Schwarz et al. (2017) [68] RCT 15 implants—15 patients.	Er:YAG 2940 nm CPS Grp: Plastic curettes+ Cotton pellets + Sterile saline/Peri—implantitis/Open flap/GBR ERL Grp: Laser Decontamination 100 mJ/pulse/10 Hz (12.7 J/cm^2^).	a/AST, BRA, CAM, ITI, KSI, REP, TSV, XIV, NI b/Mechanical or Laser decontamination.	Both grps, BOP ↓ 91.65 ± 11.08% and 66.7% at Class Ib + II defects, 88.88 ± 13.60% and 100 ± 0.0% at Class Ic + II, and 91.65 ± 11.80% and 83.30 ± 23.61% at Class Ie + II defects. SBI: Not measured. PI ↑ CPS at 83.0 mths. PD CPS ↓ + CAL ↑ 2.00 ± 0.70 mm/1.80 ± 0.70 mm at Class Ib + II defects, 3.15 ± 1.91 mm/3.25 ± 2.40 mm at Class Ic + II defects, and 1.30 ± 0.70 mm/2.25 ± 0.07 mm at Class Ie + II. ERL PD ↓ and CAL ↑ 1.17 mm/1.50 mm at the Class Ib + II defect, 1.90 ± 0.98 mm/3.60 ± 1.83 mm at Class Ic + II defects, and 0.55 ± 2.61 mm/2.30 ± 2.12 mm at Class Ie + II.	7 years ERL + CPS similar BOP ↓ CPS: 89.99 ± 11.65% vs. ERL: 86.66 ± 18.26%). CAL gains (CPS: 2.76 ± 1.92 mm vs. ERL: 2.06 ± 2.52 mm).	Combined surgical resective/regenerative therapy of advanced peri-implantitis was effective on the long-term but the clinical outcomes were not influenced by the initial method of surface decontamination.
Schwarz et al. (2017) [69] RCT 73 implants—32 patients.	CO_2_ 10,600 nm 4 Grps: Grp 1 = MD + implants, Grp 2 = MD + implants/augmented bone, Grp 3 = laser + implants, Grp 4 = laser + implants/augmented bone.	2.5 W CW. Fluence 175 Jcm^–2^ × 5 sec × 12 a/IMZ, Frialit-2 b/air-powder abrasive + CO_2_.	SBI ↓ 4 mths all groups. DIB laser no sig. Diff. Among grps during 5 yrs. PI ↓ 4 months. At 4 mths + 5 yrs stat sig. diff. between grps 1 and 3. Grp 2 and 4(GBR), stat sig diff. at 4 mths but not 5 yrs. PD no stat diff in grps 1 and 3 at 5 years. No stat diff in grps 2 and 4 (GBR) at 5 years (cf 4 mths).	5 years Clinical + X-Ray indicate CO_2_ + soft tissue resection effective against bone resorption.	1 pt (Grp 2) loss of 4 implants. 1 pt (Grp 4) loss of 4 implants. With respect to augmentation procedures, the method used for decontamination seems to play a subordinate role.
Papadopoulos et al. (2015) [70] RCT 19 patients.	DL 980 nm 2 Grps. C Grp: Cotton soaked in saline L Grp: Cotton in saline + DL PD ≥6 mm at least 1 implant + BOP + bone loss ≥2 mm Open flap debridement.	a/Not mentioned b/C Group: MD L Group: MD + Laser.	BOP 72.9% 3 mths/66.7% 6 mths (*p* < 0.05). SBI: Not measured. PI ↓ in C group at 3 mhs from 37.5 to 6.3%. No stat sig diff (*p* < 0.05) at 6 mths. CAL ↑ Laser grp 5.25 mm to 4.54 mm 3–6 mths (*p* < 0.05). PD ↓ 1.19 mm (control) + 1.38 mm (laser) (*p* < 0.05).	6 months Surgical PI Tx + flaps ↑ all clinical parameters studied. DL doesn’t have extra beneficiary effect.	Diode laser in the surgical treatment of peri-implantitis does not seem to have any additional clinical benefit.
Albaker et al. (2018) [71] RCT 24 patients.	DL 670 nm + MBO. Group 1: OFD alone Group 2:OFD and aPDT.	MBL ≥2 mm bone level 1 yr following implant or ≥3 mm PD (PA Rads) + PD ≥5 mm + BOP/open flap debridement- no GBR a/Not mentioned b/Grp 1: OFD Grp 2: OFD and aPDT BOP 6 mths ↓ 35.9–24.3% in aPDT group (*p* < 0.05). 26.5–21.6% OFD grp (*p* < 0.05). 12 mths ↓ 24.3% to 17.4% (*p* < 0.05) in aPDT and 21.6–14.8% (*p* < 0.05) in OFD grp.	SBI/DIB Not measured. PI in aPDT grp ↓ 44.7% to 21.2% (*p* < 0.05) at 6 mths and 48.3–19.5% (*p* < 0.05) in OFD group. 12 mths, PI further ↓ 21.2–16.4% (*p* < 0.05) in aPDT group and 19.5–11.6% (*p* < 0.05) in OFD group. CAL Not measured PD ↓ 6 months sig. in both groups. 5.2 mm to 3.9 mm aPDT grp (*p* < 0.05) and 5.4 mm–4.1 mm OFD group (*p* < 0.05).	12 months Both groups reduced PI, BOP, PD and MBL. However, there was no significant difference between aPDT and OFD groups over time.	Within the limits of the present RCT, it is concluded that single application of aPDT as an adjunct to OFD does not provide additional benefit in improving clinical and radiographic peri-implant parameters in peri-implantitis.
